# Extrusion-Based Additive Manufacturing with Carbon Reinforced Concrete: Concept and Feasibility Study

**DOI:** 10.3390/ma13112568

**Published:** 2020-06-04

**Authors:** Viktor Mechtcherine, Albert Michel, Marco Liebscher, Tobias Schmeier

**Affiliations:** Institute of Construction Materials, TU Dresden, 01062 Dresden, Germany; albert.michel@tu-dresden.de (A.M.); marco.liebscher@tu-dresden.de (M.L.); tobias.schmeier@web.de (T.S.)

**Keywords:** digital construction, additive manufacturing, 3D concrete printing, layered extrusion, carbon-fibre reinforcement

## Abstract

Additive manufacturing with cement-based materials needs sound approaches for the direct, seamless integration of reinforcement into structural and non-structural elements during their fabrication. Mineral-impregnated Carbon-Fibre (MCF) composites represent a new type of non-corrosive reinforcement that offers great potential in this regard. MCF not only exhibits high performance with respect to its mechanical characteristics and durability, but it also can be processed and shaped easily in the fresh state and, what is more, automated. This article describes different concepts for the continuous, fully automated integration of MCF reinforcement into 3D concrete printing based on layered extrusion. Moreover, for one of the approaches presented and discussed, namely 3D concrete printing with MCF supply from a continuous, stationary impregnation line and deposition of MCF between concrete filaments, a feasibility study was performed using a gantry 3D printer. Small-scale walls were printed and eventually used for the production of specimens for mechanical testing. Three-point bend tests performed on two different beam geometries showed a significant enhancement of both flexural strength and, more especially, deformability of the specimens reinforced with MCF in comparison to the specimens made of plain concrete.

## 1. Introduction

In recent years, Digital Concrete Construction (DCC) has increasingly attracted the attention of the construction industry and research groups. Several pilot projects have already demonstrated the high potential of the new digital production technologies [1,2,3]. In the framework of additive manufacturing (AM) with concrete, often referred to as 3D concrete printing (3DCP) as well, techniques based on layered extrusion seem to be at the present stage the most promising approach with respect to both its economic feasibility and to its prospective use in construction practice [4,5]. While considerable progress has been achieved in printing with fine mortar [3,6] and real concrete [7], the integration of reinforcement—needed in most concrete structures—remains a major challenge [5,8,9,10].

As yet, the solutions frequently used suggest the discontinuous placement of steel bars between individual concrete layers [11] or printing concrete formwork and placing conventional steel reinforcement into it, followed by filling the formwork with vibrated or self-compacting concrete; see, for example, [12]. Pre-tensioned concrete elements can be realised using the latter approach as well [3]; alternatively, unbounded pre-stressing of printed, hardened concrete elements can be applied [13]. In all these cases, the placement of reinforcement is a separate production step delivered in a conventional manner. However, there are some suggestions for automating this step by assembling prefabricated reinforcement elements [13,14] or by applying AM techniques [15].

Few publications have addressed the integration of reinforcement directly into the 3D-printing process. The most straightforward approach is working with short, dispersed fibres so that the reinforcement is part of the composite material deposited by extrusion [16,17,18,19,20]. While the performance of modern fibre-reinforced concretes such as strain-hardening cement-based composites (SHCC) is indeed remarkable [21], short fibres cannot replace continuous reinforcement elements in most structural applications.

Another approach is to equip the concrete printhead with a device that automatically places steel wire immediately in front of the nozzle. Then, a concrete filament that covers the wire is extruded through the nozzle [22,23,24,25]. However, due to their smooth surface, steel wire or wire strand bundles form only very weak bonds with the surrounding concrete. The use of such reinforcements also limits the geometric freedom of AM due to the reinforcements’ relatively high flexural stiffness. Furthermore, corrosion protection might be an issue if conventional steel is used instead of stainless steel. Lim et al. used a geopolymer (GP) concrete modified by short polyvinyl alcohol (PVA) fibres instead of a cement-based matrix in combination with steel cable [23]. In bending tests, this combination led to an increase in maximum bending force of up to 290% when compared to specimens without steel cables. However, due to the poor bond between the cable and the GP matrix, pronounced pull-out behaviour was observed.

A promising alternative concept suggested by Marchment and Sanjayan [26] uses instead of wire a strip of textile reinforcement wound on a carrier fixed to the printhead. The textile is encapsulated by the deposition of concrete layers on both of its sides using a special forked nozzle. While the freedom of form is limited in this case also, to some extent, reinforcing action in the vertical direction can be realised in addition to that in the horizontal direction due to the purposeful overlapping of the textile’s strips.

The article at hand describes a new approach formulated by this article’s first two authors and soon to be patented. Said approach promises to overcome the above-mentioned drawbacks and restrictions related to the use of steel cable or strips of textile while enabling both horizontal and vertical reinforcement of printed concrete elements. This technology builds on a novel composite, i.e., Mineral-impregnated Carbon-Fibre (MCF), developed in recent years at the Institute of Construction Materials of the TU Dresden [27,28,29]. The use of carbon fibres (CF) as reinforcement for concrete offers considerable advantages in comparison to steel bars: CF do not corrode, and they have a much lower specific weight while yielding considerably higher tensile strength; see as an example [30]. As a result, thinner, more sustainable and cost-efficient structures can be produced without trade-offs at the expense of durability and load-bearing capacity [30,31]. Note that CF rovings always need to be impregnated with a binder before its use in order to ensure the force transfer between individual filaments and bond of reinforcement to concrete matrix. In contrast to conventional textile reinforcements, where the carbon–fibre roving is impregnated with a polymer matrix [32], a micro-sized, mineral-based suspension is used as the impregnation material for MCF. One or multiple rovings can be impregnated inline before integration into additive manufacturing with concrete. Multiple rovings can be subsequently processed individually or bundled into thicker strands [33].

In prior publications, the impregnation technology was presented, and the mechanical performance of this novel type of reinforcement was investigated [27,28]. In comparison to polymer-bound carbon-fibre reinforcement, MCF exhibits superior bonding to concrete. In the case of MCF, sufficient bond strength was measured even at temperatures up to 500 °C [28,29]. The new reinforcement is also less expensive and environmentally friendlier in comparison to the polymer-bound version. However, of particular interest is the fact of MCF’s considerably increasing technological flexibility, especially with respect to emerging automated production approaches. Mechtcherine et al. [33] provided several examples for the automated manufacturing of reinforcement systems made of MCF: one-dimensional elements such as bars and strips, two-dimensional reinforcements in the form of mats, and three-dimensional cases as examples of reinforcements for a balcony and shell elements. The work demonstrated that MCF can be easily formed in the unhydrated state, the minimum bending radii are very small, so that any desired shape can be realised. This article suggests several original approaches for integrating MCF directly into the 3D concrete printing process. The main challenge is to implement the impregnation process of CF in the process chain of 3D concrete printing. The suggested approaches vary in the type of impregnation process (stationary or mobile), in the way the MCF is supplied to the printhead (one-step or two-step process), and in the solution for integrating the MCF into concrete (directly into the printed concrete filament or between concrete filaments). Furthermore, original solutions for the placement of reinforcement out of the horizontal plane and for variation of the degree of reinforcement in printed concrete are proposed. Finally, a feasibility study was conducted with one selected concept, and the results of mechanical testing are reported here, showing the high potential of additive manufacturing technologies with MCF-reinforced concrete. 

## 2. Concepts for a Direct Implementation of MCF Reinforcements in 3D Concrete Printing

The implementation of MCF reinforcement directly into the additive manufacturing process requires sound solutions for the continuous impregnation of carbon roving, the transport/supply of the roving to the printhead, and finally the integration of the reinforcement into the concrete. Various processing techniques are applicable and are presented and discussed in this section. Note that the approaches suggested can be realised with different manipulators for moving the printhead. In the following, a gantry-based manipulator is shown as the base, but the technology can be used with other robotic systems as well. Note that gear dimensions are not provided in this article on purpose. Generally, the presented setups can be scaled to any size needed to meet the requirements of particular application scenarios.

### 2.1. MCF Production and Supply to the Printhead

#### 2.1.1. Stationary Impregnation Process

**Figure 1:**
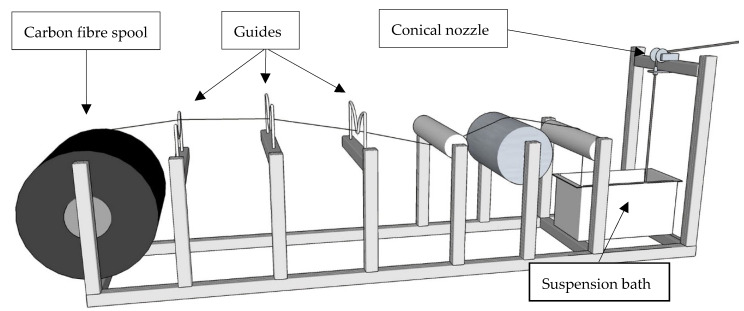
Stationary yarn impregnation line.

High-quality impregnation of the entire carbon-fibre roving with the mineral-based suspensions is a prerequisite for any processing technique presented in this article. This ensures sound interaction among the individual CF filaments and very good bonding of the yarn with the concrete matrix. Figure 1 shows schematically an appropriate setup for continuous inline impregnation.

Firstly, a carbon-fibre roving is uncoiled from the spool, levelled, and guided towards a suspension bath. At this stage, pre-treatments such as plasma modifications [27] or prewetting can be easily applied; this subject is not addressed here. Then, the roving is guided through a suspension bath, a padder with several assembled rolls. The rolls, usually three or five, enable the fanning out of the yarn by repeated deflection, which enables the very nearly complete penetration of the mineral fines among the several thousand CF filaments. Subsequently, the freshly impregnated yarn is guided through a conical nozzle, which assists in removing the surplus suspension and shaping the MCF. MCF produced in this way can be supplied to the printhead in three different ways, as presented in the following sections. 

#### 2.1.2. MCF Supply from a Spool (Two-Step Process)

In this approach, the production of MCF and the actual 3D concrete printing are two successive steps with a certain time gap in between. Although not integrated in a continuous process, this approach has some advantages. It excludes potential collisions of the guided MCF with the printhead moving along the path, thus reducing drastically the possibility of process errors. Furthermore, this approach offers the highest flexibility in respect of the use of various robotic systems and the printing of complex geometries.

**Figure 2:**
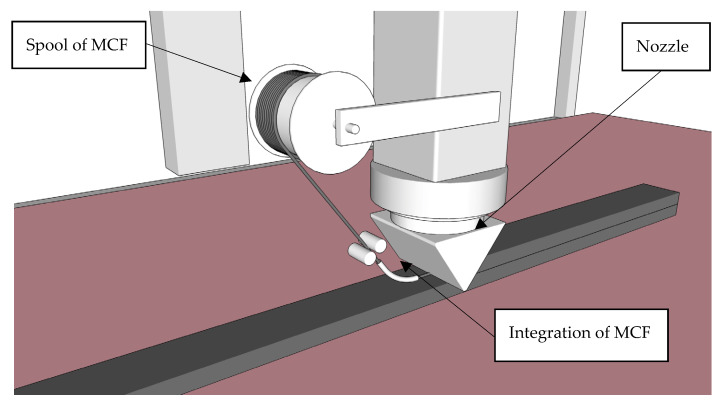
3D concrete printing with Mineral-impregnated Carbon-Fibre (MCF) supply from a spool.

In the first step, MCF roving is produced as described in Section 2.1.1; see also Figure 1. After leaving the nozzle, it is wound onto a spool. In this process, the roving’s cross-section changes its cross-sectional shape from circular to elliptical due to the slight lateral compression induced by the winding. Then, this spool is attached directly to the printhead; see Figure 2. During printing, the MCF is uncoiled and supplied to be integrated either in a concrete filament or between concrete filaments; these options are presented in Section 2.2. Depending on the reaction speed of the binder in the impregnating suspension and chemical admixtures used, the spool can be used for several hours. However, dehydration of the MCF should be avoided.

#### 2.1.3. MCF Supply from a Continuous, Stationary Impregnation Line (One-Step Process)

**Figure 3:**
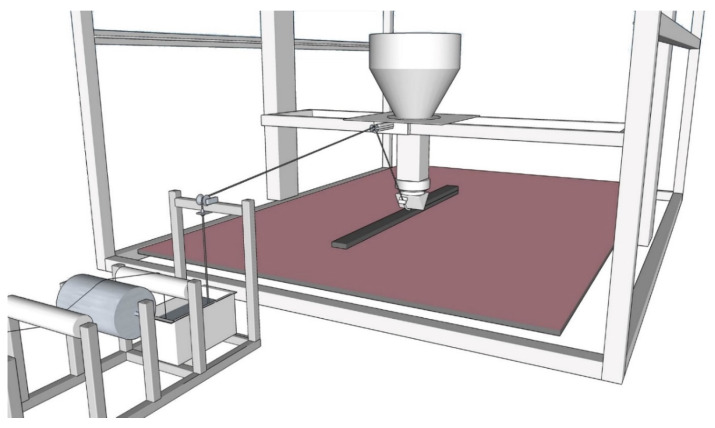
3D concrete printing with MCF supply from a continuous, stationary impregnation line.

As shown in Figure 3, in this approach, the CF yarn is firstly impregnated in place and aligned next to the printer. After leaving the conical nozzle, MCF is guided to the printhead and integrated into the layered extrusion process, as detailed in Section 2.2. This continuous process ensures a fully “fresh-in-fresh” integration of MCF into the concrete matrix and, therefore, the highest degree of compatibility of both materials. The speedy integration of the impregnated yarn into the concrete allows for perfect curing conditions for the MCF and the best possible bonding properties between both constituents. Moreover, the continuous production process is beneficial as regards quality management and economic feasibility. However, as pointed out above, direct feeding of the printhead with MCF gives rise to some drawbacks with respect to the flexibility of the entire process and to the printing of complex geometries.

#### 2.1.4. MCF Supply from a Mobile Impregnating Device (One-Step Process)

**Figure 4:**
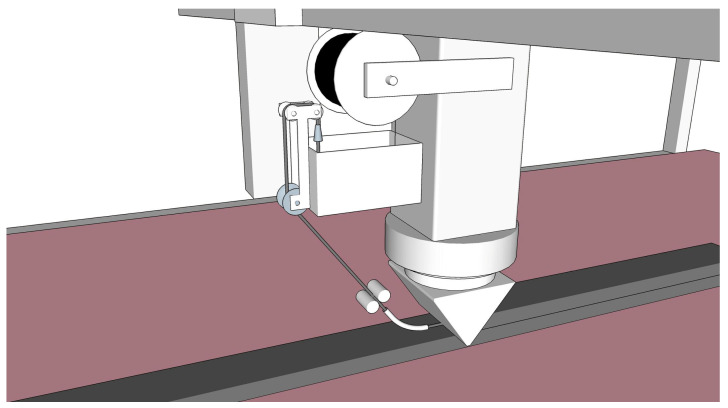
3D concrete printing with MCF supplied from a mobile impregnating device.

A further option is the impregnation of CF yarn(s) by means of a compact device attached directly to the printhead; see Figure 4. Obviously, the capacity of both the CF spool and suspension bath are considerably lower in comparison to the stationary MCF production line. This means interruptions in the 3D printing process when middle and large size elements are to be produced. Moreover, options for stretching and initial alignment of the carbon filaments as well as for pre- and post-treatment of the reinforcement are limited. However, the approach has its strengths, too, since it combines the key advantages the other two techniques: It offers both the direct incorporation of freshly impregnated CF roving into the 3D concrete during printing and a high level of geometrical freedom and process flexibility.

### 2.2. Integrating MCF into Concrete Elements

To integrate MCF reinforcement into printed concrete, two different ways are conceivable within the framework of extrusion-based 3D printing: (1) integration of the MCF directly into the printed concrete filament, and (2) deposition of the MCF onto a printed concrete filament and overprinting it with the subsequent concrete filament. Additional requirements within both approaches can be (1) the free start and stop of MCF supply and integration into concrete and (2) an adjustable degree of reinforcement. Obviously, these requirements increase the level of sophistication of the printing system but increase its flexibility and the overall efficiency in the purposeful use of the reinforcement. 

#### 2.2.1. Integrating MCF into the Concrete Filament

**Figure 5:**
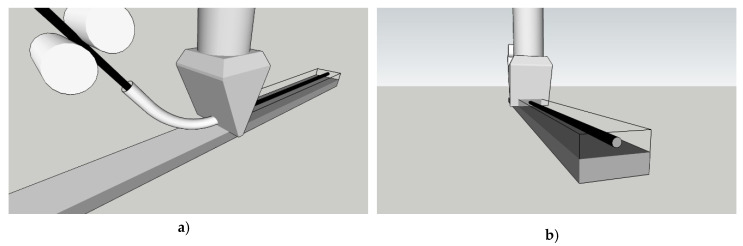
Integrating MCF into concrete filament: (**a**) supply of MCF yarn to the nozzle, and (**b**) cross-section of the printed layer with incorporated MCF reinforcement.

The integration of reinforcement directly into the concrete filament is an approach pursued recently in several research projects [22,23,26]. Flexible reinforcement is inserted into a nozzle through an opening on its reverse side, while the obverse side of the nozzle shapes the concrete filament with the integrated reinforcement. This approach appears beneficial also in integrating the MCF reinforcement, since it promises better bonding between reinforcement and concrete in comparison to the option in which the MCF is deposited between concrete layers. Figure 5 illustrates the setup as envisaged. The MCF is supplied via two rollers rotating in opposite directions and guided through a tube inserted on the backside of the nozzle. There are also limitations on such a setup: (1) deposition of reinforcement without concrete is not possible or at least problematic, and (2) the placement of reinforcement is possible only in parallel with the printed layers. The printing scenario shown as an example in Figure 7a,b can hardly be realised when the MCF is integrated directly into the concrete filament.

#### 2.2.2. Integrating MCF between Concrete Filaments

**Figure 6:**
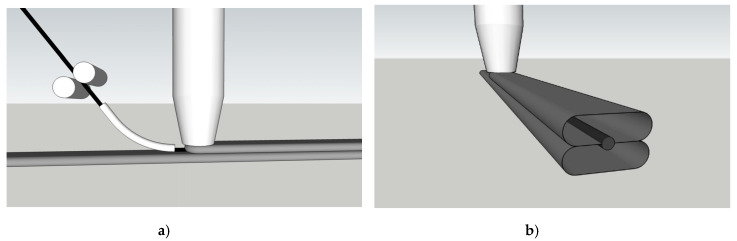
Integrating MCF between concrete filaments: (**a**) yarn supply scheme, and (**b**) cross-section of two printed concrete layers with MCF reinforcement in between.

**Figure 7:**
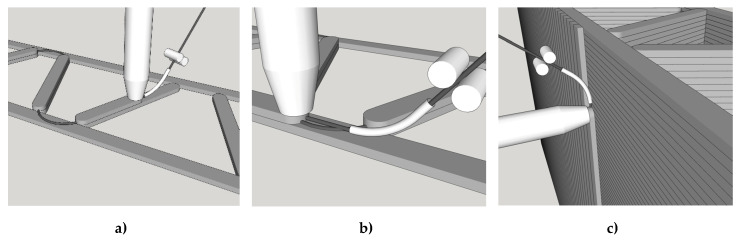
Implementation aspects for integration of MCF reinforcement between concrete layers: (**a**) continuous deposition of MCF while concrete is deposited selectively, (**b**) creating an overlap of MCF yarns, (**c**) vertical deposition of MCF and concrete.

In the second approach, the MCF reinforcement is placed between two subsequently printed concrete layers. The first part of the setup is as described above. However, the tube channelling the MCF does not insert the yarn into the nozzle, but rather deposits it in front of the nozzle just before it passes the same spot. While the MCF reinforcement is being placed, the previously printed concrete filament acts as a substrate; then, the roving is immediately covered by the following printed concrete layer extruded by the printhead; see Figure 6. It is noteworthy that the fresh MCF strand may be slightly compressed in the process and hence change its initial circular cross-sectional shape to become elliptical. The first approach, shown in Figure 5, in comparison to the second approach, as seen in Figure 6, offers both advantages and drawbacks. The main advantage is that the MCF can deposited also, indeed independent of the concrete. This facilitates the manufacture of elements with complex geometries and the specific reinforcement arrangements. The entire process is more flexible, especially if a nozzle with a vertical discharge direction is used; see Figure 6; Figure 7. On the negative side, a weaker bond between reinforcement and concrete is to be expected. Potentially, depending on the printing regime and rheological properties of concrete in the first place, the reinforcement in the interlayer joint can even weaken the bond between these concrete layers.

#### 2.2.3. Further Implementation Aspects

Concrete structures usually require reinforcements in all three spatial directions to deliver adequate load-carrying capacity in relevant loading scenarios. While no fully automated production of concrete elements reinforced in all three spatial directions has been realised so far, the suggested approaches of integrating MCF into 3D concrete printing do bring the additive technology close to this goal. 

The possibility of depositing reinforcement in the x–y plane is obvious for both approaches presented in Section 2.2.1 und Section 2.2.2. However, integrating MCF into the concrete filament excludes the option of a direct contact/overlap of reinforcing yarns/strands, while establishing such overlaps is very possible when the reinforcement can be deposited independently of the concrete, i.e., in the case of MCF integrated between concrete filaments. Note here that in many instances, overlaps of reinforcement segments are essential to achieving optimal load-bearing structural elements. An example of such overlaps is shown in Figure 7a,b for a wall with longitudinal and shear reinforcements. To realise such structures, the interruptions in concrete flow need to be well controlled, wherein the MCF is continuously supplied and deposited; see Figure 7a. The MCF already deposited is covered by a concrete filament in the following processing step; see Figure 7b. As a result, full reinforcement in multiple directions within a plane with sound interaction of individual yarns/strands can be achieved. 

The placement of reinforcement out of the horizontal plane is possible with both approaches while the limitations concerning the possibility of reinforcement overlaps remain in the case of integrating the MCF directly into the concrete filaments. On the one hand, inclined printing paths can be created, as already demonstrated for 3D printing without reinforcement, e.g., by purposely varying the thickness of concrete layers [34] or by depositing concrete filaments on a curved platform [35]. On the other hand, once several (reinforced) concrete layers are deposited and the wall has gained sufficient strength, inclined or even vertically reinforced concrete layers can be printed on its surface. Figure 7c shows an example of placing vertical MCF reinforcement eventually covered by concrete filaments. 

Finally, it should be noted that the degree of reinforcement in printed concrete can be varied by:

- deposition of one, two, or several rovings next to each other,
- bundling of several rovings into a thicker strand before integrating them into concrete,
- use of CF yarns with different fineness,
- variation of concrete filament dimensions, etc.

The particular concrete printer setups for implementing these approaches are under development at the TU Dresden.

## 3. Feasibility Study—Materials and Methods

### 3.1. Raw Materials

MCF reinforcement is a carbon yarn impregnated with a mineral-based suspension. In this study, SIGRAFIL C T50-4.4/255-E100 produced by SGL Group (Wiesbaden, Germany) and containing approximately 50,000 individual CF filaments was used. Table 1 gives further technical data. To facilitate a high degree of impregnation, a mixture of ultra-fine binders is needed to make the suspension. In particular, the micro-cements Mikrodur R-X and Mikrodur P-U produced by Dyckerhoff (Wiesbaden, Germany) with d95 of maximum 9.5 µm were used with the silica suspension Centrilit Fume SX and a superplasticizer MSH flüssig produced by MC Bauchemie (Bottrop, Germany). The water-to-binder ratio of 0.8 and a superplasticizer-to-binder ratio of 3.6% were chosen to attain favourable rheological properties of the suspension. Table 2b gives the exact composition of the impregnating suspension.

The composition of fine-grained concrete used for 3D printing in this investigation was adapted from the previous studies at the TU Dresden [7,36,37]. Note that the authors followed the premise of using as high aggregate fraction and correspondingly as large a maximum aggregate size as possible for the chosen filament cross-section, cf. Section 3.3, and the size of specimens produced for mechanical testing, cf. Section 3.4. Thus, a composition with a maximum aggregate size of 4 mm was selected. The testing and adjustment of the rheological properties of fresh mixture for 3D printing was performed in accordance of the requirements described, in e.g. [7,38]. Table 2a details the concrete composition. The binder mix consisted of CEM I 52.5 R (OPTERRA Zement GmbH, Werk Karsdorf, Germany), fly ash Steament H-4 (STEAG Power Minerals GmbH, Dinslaken, Germany), and silica suspension EMSAC 500 SE (BASF, Friedrichshafen, Germany). Additionally, a polycarboxylate ether (PCE)-based superplasticizer (BASF, Germany) was used to adjust the rheology of the fresh mixture. 

### 3.2. Fabrication of the MCF and Concrete Mixing 

**Figure 8:**
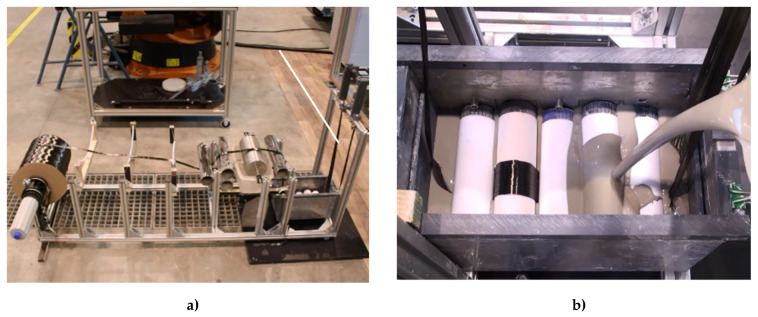
Fabrication of MCF: (**a**) entire setup, and (**b**) five-roller padder used for impregnation.

Mixing of the impregnating suspension was conducted in two steps. Firstly, all ingredients were premixed using a standard kitchen blender. Subsequently, a T 50 digital ULTRA-TURRAX® was used for two minutes at 7000 rpm to disperse all mineral fines completely. The impregnation process was executed according to Section 2.1.1. Figure 8a shows the entire line used, including a five-roller padder (Figure 8b). After impregnation, the yarn was shaped by means of a conical nozzle with an inner diameter of 3.8 mm.

The 3D printable concrete was produced using a single-shaft pan mixer. Solid materials were first mixed in the dry state for two minutes, followed by adding the fluid components. The initial rotational speed of 25 rpm was increased to 45 rpm after one minute and subsequently maintained for four minutes.

### 3.3. 3D Printing of Carbon-Reinforced Concrete Elements

3D concrete printing was performed with MCF supply from a continuous, stationary impregnation line, thus using the one-step process described in Section 2.1.3 and MCF integration between concrete filaments as presented in Section 2.2.2. After leaving the shaping nozzle, the MCF was fed directly to the gantry robot described in detail in [7]. The printhead contained an Archimedean screw mechanism to extrude the concrete. The extruder was supplied from a concrete container, which was designed as a truncated cone with a capacity of 60 L. The consistent feeding of the extruder with concrete was supported by helical metal blades. The cross-section of printed filaments was 85 mm × 27 mm. The printing speed was 20 mm/s, and the flow rate was set at 2.75 L/min.

The gantry robot and all associated motors and sensors were controlled using a customized software utilizing LUA scripting embedded in a graphical user interface (GUI). Instructions for printing the specimens, i.e., coordinates, velocities, directions, and material flow parameters, were predefined in the scripts or manually controlled through the GUI.

**Figure 9:**
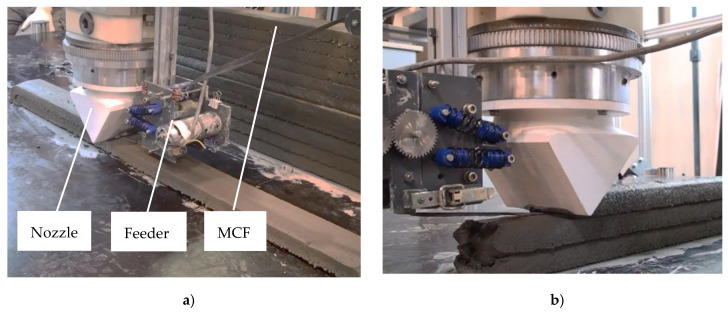
Printing of carbon reinforced concrete walls: (**a**) overview and (**b**) a detailed view of the nozzle with the MCF feeder.

**Figure 10:**
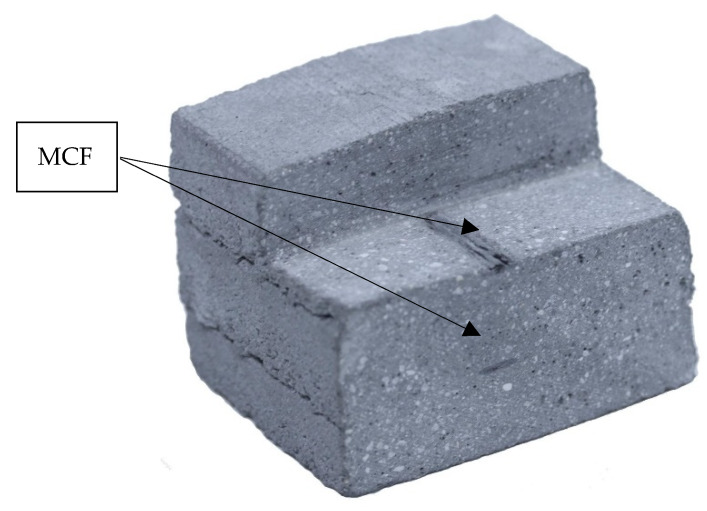
Cut-out of a printed wall showing MCF reinforcement between concrete layers.

The MCF supply was arranged in such a way as to enable the nearly stress-free deposition of the reinforcement. For this purpose a roller-based feeder for the backside of the nozzle was developed; see Figure 9a,b. The MCF was deposited automatically onto the previously printed filament and immediately covered by the next concrete layer. By repeating this process several times, a carbon-reinforced concrete wall was fabricated. For the testing program presented in this article, walls of 11 layers were produced. The time gap between layers was approximately 3 min. After printing, the specimens were covered with plastic foil for one day. Figure 10 shows the cut-out of a printed wall with an MCF reinforcement well integrated between the concrete layers. It also can be seen that the cross-sections of the MCF are not circular, as shaped by the conical nozzle, but flattened into an elliptical shape due to the immediate covering with concrete.

### 3.4. Specimen Preparation and Mechaniical Testing

**Figure 11:**
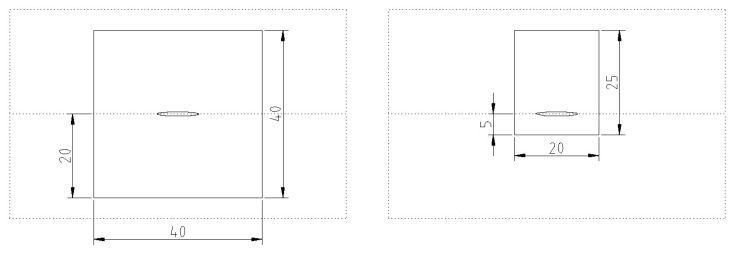
Cross-sections of the specimens for mechanical testing: (**a**) 40 mm × 40 mm with MCF positioned centrally and (**b**) 25 mm × 20 mm with reinforcement positioned in the tension zone.

Six days after the printing process, the MCF-reinforced concrete was prepared for mechanical testing. To assess the effectiveness of this novel reinforcement, beam specimens of two different geometries were sawn out of the printed walls: (1) dimensions of 120 mm × 40 mm × 40 mm, with the MCF reinforcement located centrally; see Figure 11a; and (2) dimensions of 120 mm × 20 mm × 25 mm, with the MCF positioned in the tension zone; see Figure 11b. The purpose of using two different specimen geometries was to attain two different degrees of reinforcement in concrete (the fineness of the reinforcing yarn remained constant). Note that in both geometries, the reinforcement is located 20 mm below the top of the specimen; thus, the degree of reinforcement of the second specimen type is twice as high as for the first specimen type. In addition, reference samples without reinforcement were produced for both geometries. 

**Figure 12:**
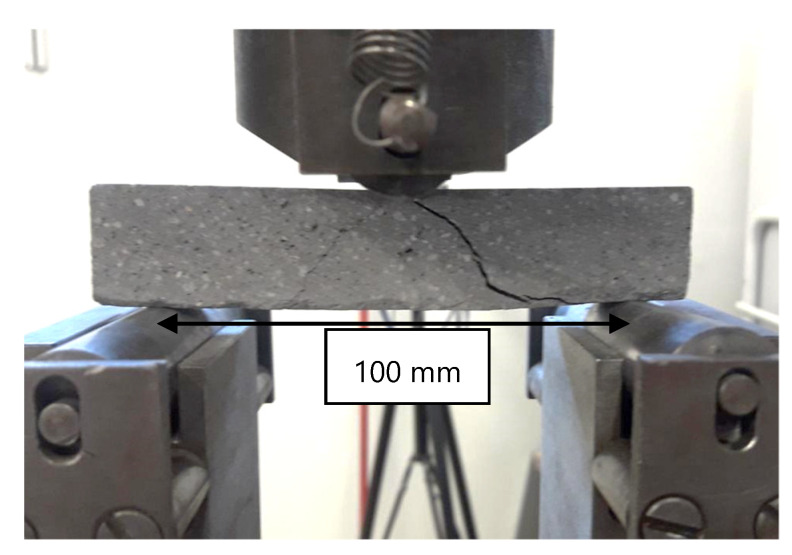
Three-point bend test on an MCF-reinforced specimen.

Three-point bending tests were performed at an age of 7 days using a path-controlled Zwick-Roell 1200 with a load cell of 600 kN; see Figure 12. The span was 100 mm, and the displacement rate was 0.5 mm/s. Six specimens were tested for each parameter combination.

## 4. Feasibility Study: Results and Discussion

### 4.1. Speciemens with a Cross-Section of 40 mm × 40 mm and Centrally Positioned MCF Reinforcement

**Figure 13:**
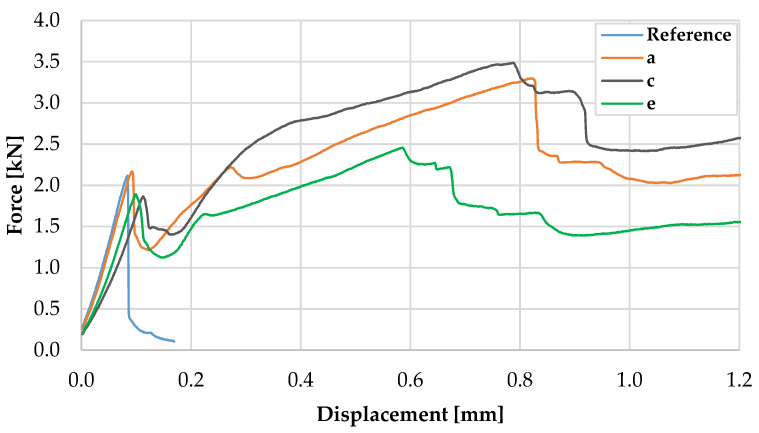
Representative force–displacement curves for specimen with a cross-section of 40 mm × 40 mm.

Figure 13 presents the representative force–displacement curves for 40 mm × 40 mm specimens with and without MCF reinforcement. At the beginning of loading, the curves exhibited an almost linear course up to a mean force value of 1.94 kN at a displacement of approximately 0.9 mm; see also Table 3. In this range, the behaviour of the specimens with and without reinforcement was almost identical. Nonetheless, the latter failed upon reaching an average maximum force of 1.96 kN, which corresponds to an average flexural strength of 4.58 MPa. In the MCF-reinforced samples, a crack formed at approximately the same stress level, followed by a drop in force down to approximately 1.1 kN. Subsequently, a pronounced increase in force was observed, which can be traced back to the mechanical activation of the MCF. The average maximum force obtained was 2.69 kN, which corresponds to an increase in load-bearing capacity or flexural strength of 38% in comparison to the plain concrete. However, much more distinct was the increase in displacement at maximum force; it rose by an order of magnitude. 

**Figure 14:**
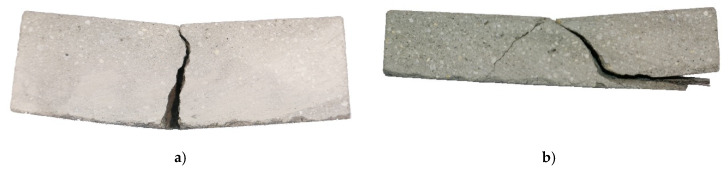
Tested specimens with cross-section of (**a**) 40 mm × 40 mm and (**b**) 20 mm × 25 mm.

Figure 14a shows a representative specimen with MCF reinforcement after testing. Only one crack was formed, indicating yarn pull-out as the failure mechanism. While partial delamination of the reinforcement from the matrix likely occurred before maximum force was reached, it can be assumed that the delamination was complete, and the beginning of the actual pull-out coincides with a significant drop in force after reaching its maximum.

To assess the effectiveness of the reinforcement, the tensile force at failure was estimated. In doing so, the bending moment in the cracked cross-section, the middle of the beam, was expressed by the tensile force in the MCF reinforcement F_t,MCF_, compressive force in concrete F_c,C_ and the distance, or leverage, between the two forces. While the exact location of MCF reinforcement could be measured at the cracked cross-sections, the height of the compression zone was assumed to be 5% of the specimen height and the shape of compressive stress curve was assumed to be rectangular. The calculated forces F_t,MCF_ fell within the range of 2.2 to 4.6 kN, i.e., 3.1 kN on average, which corresponds to tensile stress in the carbon filaments of 1177 to 2462 MPa, i.e., 1659 MPa on average.

### 4.2. Specimens with a Cross-Section of 20 mm × 25 mm and MCF located in the Tension Zone

**Figure 15:**
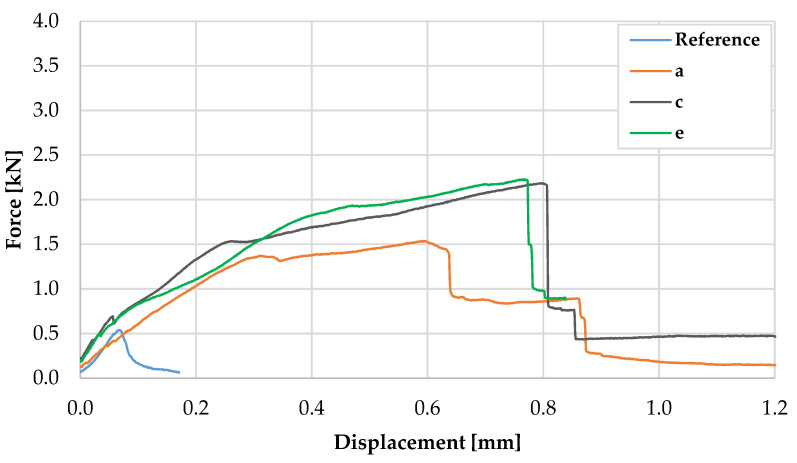
Representative force–displacement curves for specimen with a cross-section of 25 mm × 20 mm.

The flexural performance of the specimens with a cross-section of 20 mm × 25 mm and the MCF located in the tension zone differed significantly to that of the specimens with a cross-section of 40 mm × 40 mm; see Figure 15. The average maximum force of the reference specimens was 0.56 kN, which corresponds to a flexural strength of 8.42 MPa; see also Table 4. The difference in the flexural strength of the larger specimens made of plain concrete can be explained by size effects; see, e.g., [39]. For the reinforced specimens, the first crack formation was not clearly detectable, in contrast to the tests on 40 mm × 40 mm specimens. Furthermore, instead of one crack, several narrow cracks formed, which were reflected in the slight drops in the force–displacement curves with increasing deflection. The average value of the maximum force was approximately 1.9 kN, which means an increase of 238% when compared to the plain concrete, while the displacement at maximum force rose by an order of magnitude. 

The observed final failure mode was delamination and spalling of the concrete cover, which was accompanied by a yarn pull-out; see Figure 14b. Mostly due to the poorly defined failure, the calculation of the tensile force in the reinforcement F_t,MCF_ could not be performed here.

## 5. Summary and Conclusions

The authors have proposed and critically discussed various approaches to implementing Mineral-impregnated Carbon-Fibre (MCF) reinforcement in additive manufacturing by layered extrusion. These approaches vary in the mode of MCF supply to the printhead and in the fashion in which the reinforcement is integrated into the printing process as such. While the individual options presented exhibit certain advantages and disadvantages depending on the application scenario, in total, they clearly demonstrate the high flexibility and adaptability of the new technology in its prospective employment in digital construction. However, much work needs to be done to implement the approaches into real manufacturing facilities. 

The first step towards this was done with a feasibility study reported in the article. The implemented approach was 3D concrete printing with MCF supply from a continuous, stationary impregnation line and deposition of the MCF between concrete filaments. A gantry robot was used as 3D printer to produce small-scale walls, from which specimens for mechanical testing were cut out. Three-point bend tests demonstrated a significant increase in flexural strength for the specimens reinforced with MCF in comparison to the specimens made of plain concrete. An even higher impact—enhancement by an order of magnitude—was observed with respect to the deformability of the beams. In ongoing research at the TU Dresden, further approaches will be realised, tested, and presented in respect of their feasibility and reinforcing effectiveness.

In the follow-up research, several issues are to be addressed that were not in the focus of the present publication. The first aspect is the effect of the rheological properties of printable concrete on the integration process of MCF reinforcement and the bonding between MCF and concrete. Secondly, the production process of MCF itself and the mode of the MCF supply to the printhead need to be illuminated with respect to the resulting mechanical properties of the composite. In addition, the effect of the modalities of the printing process, e.g., time intervals between the layers, needs clarification. Next, deformation- and failure-behaviour should be more closely investigated for various loading modes such as bending, uniaxial tension, and shearing. Furthermore, the technology needs to be proved on complex geometries with three-dimensional reinforcement. Finally, the shrinkage behaviour and durability of MCF-reinforced printed concrete elements are to be studied. 

## Figures and Tables

**Table 1:** Technical data of carbon yarn.

Number of Filaments	50,000
Fineness of yarn [tex]	3450
Density [g/cm³]	1.8
Filament diameter [μm]	6.9
Filament tensile strength [MPa]	4400
Filament modulus of elasticity [GPa]	255
Sizing type	Epoxy
Sizing level [% by mass]	1.0

**Table 2:** Composition of: (**a**) 1 m³ fine-grained concrete and (**b**) 1 litre mineral suspension.

(**a**)		(**b**)	
CEM I 52.5 R Opterra [kg]	538	Mikrodur R-X [g]	417.5
Fly ash steament H4 [kg]	215	Mikrodur P-U [g]	417.5
Silica suspension Emsac 500 SE [kg]	162	Centritit Fume SX [g]	417.5
Sand 0.006–0.2 (BCS 413) [kg]	656	Superplasticizer MSH *flüssig* [g]	37.6
Sand 0–2 [kg]	979	Water [g]	387.5
Sand 0–4 [kg]	652		
Superplasticizer Sky 593 BASF [kg]	16		
Water [kg]	276		

**Table 3:** Results of bend tests on specimens with a cross-section of 40 mm × 40 mm.

Specimen	Reference	Reinforced Samples				
	Max. Force [kN]	First Crack [kN]	Max. Force [kN]	Increase	No. of Cracks	Failure Mode
a	2.09	2.17	3.30	69%	1	Slip
b	2.12	1.98	2.17	11%	1	Slip
c	2.21	1.87	3.49	78%	1	Slip
d	1.70	1.56	2.17	11%	1	Slip
e	1.93	1.89	2.46	26%	1	Slip
f	1.69	2.17	2.57	32%	1	Slip
Average	**1.96**	**1.94**	**2.69**	**38%**	-	-
Coeff. of var.	**11%**	**12%**	**21%**	**77%**	-	-

**Table 4:** Results of bend tests on specimens with a cross-section of 25 mm × 20 mm.

Specimen	Reference	Reinforced Samples				
	Max. Force [kN]	First Crack [kN]	Max. Force [kN]	Increase	No. of Cracks	Failure Mode
a	0.60	-	1.54	174%	2	Del.
b	0.62	0.50	1.51	169%	2	Del.
c	0.49	0.69	2.18	289%	3	Del./Slip
d	0.56	-	2.02	260%	2	Del./Slip
e	0.56	-	2.23	297%	2	Del./Slip
f	0.54	0.70	1.92	241%	1	Del.
Average	**0.56**	**0.63**	**1.90**	**238%**	**-**	**-**
Coeff. of var.	**8%**	**18%**	**16%**	**23%**	**-**	**-**
